# Determinants of marketing performance: empirical study at National Commercial Bank in Jakarta Indonesia

**DOI:** 10.1186/s40064-016-3362-3

**Published:** 2016-10-01

**Authors:** Nandan Limakrisna, Syahril Yoserizal

**Affiliations:** 1 Doctoral Management Science Program, Universitas Persada Indonesia YAI, Jl. Dipenogoro no. 74, Jakarta, 10430 Indonesia; 2 Vocation Department, LP3I, Jl. Keramat Raya, Jakarta, 10430 Indonesia

## Abstract

**Background:**

Indonesian banking industry has experienced up and down as can be seen after Pakto ‘88, in which the number of new banks grew rapidly, but after the 1997–1998 financial crisis, a lot of banks were liquidated due to the deteriorating financial condition and violation of the precautionary principles by bank management. The purpose of this research is to determine and analyze the effects of good corporate governance, information technology, HR competencies on competitive advantage and its implication on marketing performance.

**Methods:**

The method used in this research was a descriptive survey and explanatory survey with a sample size of 320 respondents, and the data analysis methods used are structural equation modeling.

**Discussion and evaluation:**

Based on the results of the research, the findings obtained from good corporate governance, information technology, HR competencies have a significant effect on competitive advantage on the performance of marketing. However, when seen in part, competitive advantage has a dominant effect on marketing performance.

## Background

Indonesian banking industry has experienced up and down as can be seen after Pakto ‘88, in which the number of new banks grew rapidly, but after the 1997–1998 financial crisis, a lot of banks were liquidated due to the deteriorating financial condition and violation of the precautionary principles by bank management.

Recovery efforts or restructuring of the banking industry have changed the ownership structure of banks and banking regulations. Many family-owned banks, have been taken over by the government and foreign owners. Similarly, some banks should be liquidated or merged. These conditions show a change in the competitive environment so that the performance of the banking industry continues to pursue improvements.

In order to create a better, healthier, and more stable banking industry, the existence of the banking structure needs to be assessed and stated in the Indonesian Banking Architecture (API). One of the pillars of the API is to create a sound banking structure. The consolidation process to strengthen the capital and the merger is expected to occur in the future in line with the API program. API has been implemented in order to build a banking industry that has a strong structure to maintain the stability of the financial sector. Consolidation policy has been conducted in addition to strengthening the financial infrastructure through the establishment of the Deposit Insurance Agency (LPS), risk management certification, and customer complaint mechanism. Another policy is to increase the level of prudential banking that refers to the international standard, the 25 basel core principles for effective banking supervision (LPI 2006). Government’s attention to the banking industry is characterized by the lack of rules regarding 78 % LDR issued by Bank Indonesia. The rule has a good goal to encourage banks to frequently provide more loans to the public.

With the attention of Bank Indonesia and the government to the banking industry, the banks in Indonesia should continue to improve their performance compared to those in some countries. However, the performance of the national public banking remains relatively stagnant or even shows no improvement and are still below under the bank some Asian countries. The performance of national public banking is still relatively low as well as in the general performance of the national banking system at the regional level in DKI Jakarta.

Based on the data from the Bank Indonesia (2011), the development of third-party funding (TPF) tends to stagnate, even in 2011, there was a decline in growth, IE growth of 18.2 % (yoy) in the second quarter of 2011, compared to first quarter in 2011 (18, 8 %, yoy). SME loan growth was relatively stagnant, even credit growth in 2011 relatively declineed and the first quarter growth in SME loans amounted to 7.9 % (yoy) to 7.5 % (yoy) in the second quarter. Hereinafter, the loan to deposit ratio (LDR) during 2010–2011 in the second quarter was still below the standard NPL Bank Indonesia (78 %).

Subanidja (2006: 15), states that the bank’s performance is indicative of the performance of the banking marketing so that the poor performance of the national public banking in Jakarta tends to be caused by poor marketing performance.

Denton (2009), suggests that the company that has a competitive advantage will be able to achieve a high performance marketing/superiority because the superior marketing performance can be achieved either through competitive advantage or comparative and cooperative advantages. Then, Ilyas (2011: 1233) state that superior marketing performance will be achieved through three advantages, namely comparative advantage, competitive advantage/competitive, and cooperative advantage. Based on the above two statements, the poor performance of the national commercial banking of the regional marketing in Jakarta tends to be caused by low competitive advantage. This is shown by the research conducted by Ratnaningsih and dan Wiguna (2010) which shows that the elements of competitive advantage such as delivery speed, quality, price levels, technology, and networks still provide low benefits while costs are high.

Lado (2007) view that the resources in terms of the competency of human resources are used for achieving the competitive advantage. Lack of national competitive advantage of regional banking in Jakarta tends to be caused by the relatively low HR competencies. Some of the central bank offices in Jakarta which demonstrate the ability of internal resources are still below the standard set by Bank Indonesia capabilities, such as lack of human resources, corporate resources, and physical resources. The poor performance of bank marketing in the Jakarta regional competitive disadvantage os also likely to be caused by a lack of human resource competencies. It can be seen from the human resources (people) that are owned by banks that reflect less business strategy built. Service industries such as banks, human resource are a very important factor for human resources that play an important roles for its client services both creditor and debtor, the use of technology tools, as well as interacting with customers.

Almajali (2011: 5) states that the implementation of information technology in the management facilitates business decision making so as to create a sustainable competitive advantage. Lutfiyahita (2011) the use of information and communication technology in the national banking sector is relatively more developed than others, but its use is still not optimal. Based on this statement, it can be said that the implementation of banking information technology is less than optimal leading to the lack of achieving competitive advantage.

Ping (2011: 10) state that the model of good corporate governance will increase the competitive advantage and performance of banking. Based on these statements, it appears that the competitive advantage and performance of the regional banking industry, especially low Jakarta, are allegedly caused by a lack of good corporate governance. This is shown by the Indonesian Banking Survey Report (2011:14) that a lot of fraud taking place in national public banks is due to the lack of good corporate governance, for instance identity fraud (29 %), internet banking and ATM fraud (21 %), fraudulent transfer of funds (19 %), collusion between employees and customers (25 %), and other corrupt practices (6 %).

In the previews research, like Denton (2009) just competitive advantage have effect on marketing performance. Lado (2007) view that just the resources in terms of the competency of human resources are used for achieving the competitive advantage and also have effect on marketing performance. Almajali (2011: 5) states that just the implementation of information technology in the management facilitates business decision making so as to create a sustainable competitive advantage, and also effect on marketing performance. Ping and Wing (2011: 10) state that just the model of good corporate governance will increase the competitive advantage and performance of banking. But Novelty in this research difference with the previews research, because in this research will test as a simultaneous effect competency, information technology, and good corporate governance on competitive advantage, also will increase marketing performance.

## Hypotheses and methodology

### Good corporate governance

Teng et al. (2011: 1181), good corporate governance (GCG) is needed to encourage the creation of a market that is efficient, transparent and consistent with legislation. The main purpose of good corporate governance (GCG) is a system created checks and balances to prevent the misuse of corporate resources and keep pushing the growth of the company (Dyah 2011: 54).

In UU No. 40 of 2007 the principles of Good Corporate Governance (GCG) should reflect on the following matters: transparency, accountability, responsibility, independence, fairness.

### Information technology

Kotler and Keller (2009: 141) to enter banking information technology services is defined as “a coordinated collection of data, system, tools, and techniques with supporting software and hardware by which an organization gathers and interprets relevant information from business and the environment and turns it into a basis for marketing action”.

Talvinen (2005: 26) argues that IT is broadly built by three major components which are Database, Hardware, and Software Systems. Database consolidates the various records that are stored in separate files in the common composite data elements that provide data for many applications (O’Brain 2005: 211). Hardware system of a hardware device that is used as a tool to assist in decision-making, including computer components, communications equipment, ATM and other computer system components (Ali 2009). Software systems are available for the end user, the main categories of system software and application software. Software systems perform information processing jobs for end users and software application to manage and support the operation of computer systems and networks (O’Brain 2005: 155).

### HR competencies

Paula (2011: 384) argue that the competence of HR is a human resources expertise that includes technical, strategic, organizational, interpersonal, and personal management.

Vos (2011: 5) define human resource competencies, *“*an underlying characteristic of an individual that is causally related to criterion-referenced effective and/or superior performance in a job or a situation*”.*

Hudson and Radu (2011) state that HR competence consists of two dimensions, namely attitude and behavior competency.

### Competitive advantage

Bennett and Smith (2002: 75), competitive advantage achieves excellence through superior customer value by creating a competitive strategy to achieve profitability and growth.

Hsieh et al. (2005: 12) state that “A competitive advantage involves a series of systematic and related decisions that give a business a competitive over other businesses. The concept of business competitive advantage is primarily derived from Porter’s classifications of generic strategies.” The business strategy of a company is geared to win the competition in the target market. A competition will be won if the manufacturer is able to create a business strategy that has a competitive advantage (competitive advantage).

Suryaningsih and Abdul (2010: 14), an indicator of the competitive advantage of banks is as follows: Delivery Speed, Quality, Culture, Price Level, Customer Know-how, Technology, Networks, R & D, Management, Contract, and Safety.

### Marketing performance

Basically, the sale is one of the performance marketing which is the level of work achieved by the company during the period, compared to the operational objectives, standards and criteria that have been set previously.

Pribadi and Kanai (2011) argue that performance marketing is an achievement that earned the company from marketing activities are done. Performance marketing is more precisely measured through the customer’s perspective, namely customer satisfaction, customer profitability, and the acquisition of new customers.

**Fig. 1:**
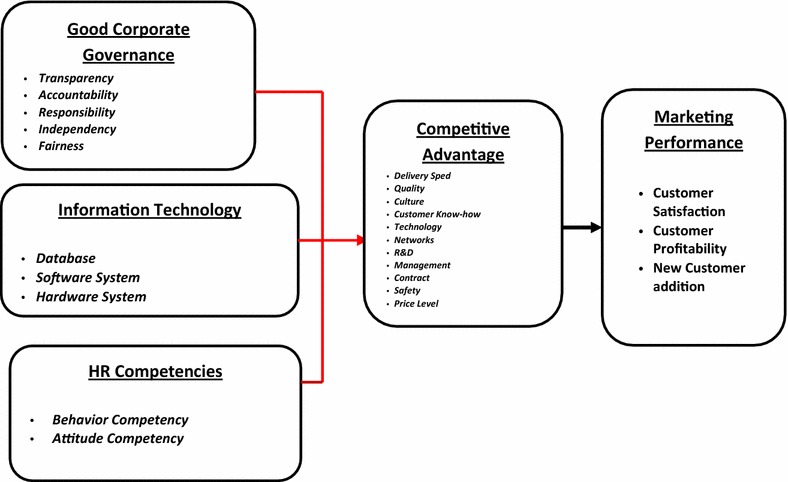
Conceptual model

Based on the framework above, conceptual research model can be described as in Fig. 1.

### Hypothesis

H1: There is an influence of good corporate governance on competitive advantage
H1a: There is an influence of transparency on competitive advantage
H1b: There is an influence of accountability on competitive advantage
H1c: There is an influence of responsibility on competitive advantage
H1d: There is an influence of independency on competitive advantage
H1e: There is an influence of fairness on competitive advantage
H2: There is an influence of information technology on competitive advantage
H2a: There is an influence of database on competitive advantage
H2b: There is an influence of software system on competitive advantage
H2c: There is v influence of hardware system on competitive advantage
H3: There is an influence of HR competence on competitive advantage
H3a: There is an influence of behavior competency on competitive advantage
H3b: There is an influence of attitude competency on competitive advantage
H4: There is an influence of competitive advantage on marketing performance

### Methods and procedure

The nature of this research is descriptive and verification, and the research method used was a descriptive survey method and explanatory survey. The type of investigation in this study is causality. The unit of analysis in this study is the organization, in which a national public banking unit of observation is the manager of the National Bank in Jakarta. Time frame in this study is cross-sectional, i.e. the information of most of the population (the sample of respondents) was collected directly from empirically location in order to know the opinion of the major population towards the object being studied.

Sources of data in this research are a secondary data source of documentation or reports which are available to the agency. Primary data in the form of good corporate governance, information technology, human resource competencies and competitive advantages, and performance marketing is sourced from the Bank Manager.

The total population in this study was 90 commercial banks, and the sample size using the formula Slovin (1998; 49) is 48 commercial banks, and the number of respondents who used the unit study using a stratified random sampling method was of 320 respondents.

The method of analysis used in this study is the analysis of structural equation modeling (SEM) by using processing software Lisrel 8:30. In the SEM analysis method, statistical estimation was individually tested using the t test. Through the output path diagram, statistical t value, *t* test results confirmed Lisrel with the complete test error rate set at 5 %. In addition to individual, SEM also tested the proposed model as a whole, namely through conformance test model (goodness of fit statistics).

## Result and discussion

Based on Table 1, three sizes obtaining suitability index models have a good fit, the GFI, RMSEA, and AGFI. Other suitability index models are under either measure compliance, but still within the scope of the suitability of the marginal (marginal fit). Marginal fit is the congruence condition measurement model under the criterion measure of absolute fit, incremental fit, but can still be passed on further analysis, because it is close to the size criteria of good fit (Hair, Andersen, Tatham, and Black 2006: 623).

**Table 1:** Model suitability

GOF indicator	Expected result	Estimation result	Conclusion
*Absolute fit*			
GFI	GFI > 0.90	0.91	*Good Fit*
RMSEA	RMSEA < 0.08	0.03	*Good Fit*
*Incremental fit*			
NNFI	NNFI > 0.90	0.79	*Marginal Fit*
NFI	NFI > 0.90	0.79	*Marginal Fit*
AGFI	AGFI > 0.90	0.93	*Good Fit*
RFI	RFI > 0.90	0.76	*Marginal Fit*
IFI	IFI > 0.90	0.82	*Marginal Fit*
CFI	CFI > 0.90	0.82	*Marginal Fit*

Source: results of treatment with LISREL 8:30

Based on the results of the analysis with Lisrel 8:30 embodied in the unity hypothesis (H1) to the ninth hypothesis (H9) are depicted in Table 2:

**Table 2:** Hypothesis testing

Hypothesis	Variable	Standardize coefficients	t value
H1	Good corporate governance *→* competitive advantage	0.28	*2.78*
H1a	Transparency **→** competitive advantage	0.24	*2.56*
H1b	Accountability **→** competitive advantage	0.27	*2.77*
H1c	Responsibility **→** competitive advantage	0.31	*3.17*
H1d	Independency **→** competitive advantage	0.35	*3.35*
H1e	Fairness **→** competitive advantage	0.30	*3.12*
H2	Information technology *→* competitive advantage	0.45	*4.44*
H2a	Database **→** competitive advantage	0.29	*2.79*
H2b	Software system **→** competitive advantage	0.25	*2.79*
H2c	vHardware system **→** competitive advantage	0.21	*2.12*
H3	Human resource competencies *→* competitive advantage	0.26	*3.75*
H3a	Behavior competency **→** competitive advantage	0.38	*3.41*
H3b	Attitude competency **→** competitive advantage	0.29	*2.79*
H8	Competitive advantage **→** marketing performance	0.70	*10.33*

Source: results of treatment with LISREL 8:30

Significant t values are in italics (t > 1.96)

Based on the above results, the national public banking in Jakarta will be able to improve marketing performance, especially in customer profitability (Y13) if the company has a competitive advantage, especially in high technology (Y5), while the competitive advantage in the company’s technology will be superior if the company has a reliable information technology system, especially in applications (software) (X8) and have good corporate governance, especially in the independent (X4) in implementing the precautionary principle and supported by human resources especially in the behavior of high competency (X9).

Then, based on the perception of the general manager of the national banking system in Jakarta, corporate governance (GCG) is still relatively poor, especially in such elements that provide complete and accurate information, the level of ease for access by stakeholders according to their rights in giving opportunity to all stakeholders to provide input/opinions, and in providing access to information in accordance with the principle of openness. Based on this, the national commercial banks in Jakarta are demanded to pay more attention to and improve the implementation of good corporate governance (GCG). Therefore, competence provides complete and accurate ease of access to stakeholders according to their rights, and opportunities provide constructive input from stakeholders. This can be done by increasing the ability of the application system and HR competences. Thus, openness, accountability, and fairness of the National Banking can be improved (Permata Budi Asri 2011; Ping et al. 2011; Teng et al. 2011).

Information technology (IT) of a national public bank in Jakarta is still relatively low, especially in the elements of security such as ATM availability, transaction availability through internet and phone banking through transaction availability, completeness featured on ATMs, and the completeness of the features on internet banking. Therefore, it is necessary that national public banking in Jakarta be able to improve their skills in the use of information technology to provide easiness to customers, debtors, and other stakeholders in the transaction. The ability to use new information technology is only held by four (big four) banks. (Almajali 2011; Hsieh et al. 2005; O’Brain 2005).

HR competencies owned by national commercial banks in Jakarta still need attention, and these elements relate to feelings of being needed by others, easily satisfied, being tired, waiting for free time, the tendency to do something, the tendency to be lazy, and waiting for retirement. This suggests that the attitude of human resource competency owned by banking companies needs to be improved so as to contribute to the companies. The steps can be carried out by the national public banking in Jakarta through training, soft skill development, proper rotation system, career development, and transparent and non-discriminatory promotion system (Vos et al. 2011; Paula et al. 2011; Hudson et al. 2011).

Competitive advantage in the national public banking in Jakarta is still relatively low, especially on elements such as the speed of product offerings or services to customers, the technology used, extensive networks, research and development activities, and the level of costs offered. This shows that the speed in product offerings or customer service needs to be improved or repaired, so that the company’s products can be enjoyed by the banking customers with superior service. Then, from the aspects of network technologies and the development and improvement, customers, debtors and other stakeholders need transaction with ease (Bennett et al. 2002; Suryaningsih and Abdul 2010).

National public banking marketing performance in Jakarta is still relatively low, especially on the elements in improving the total customer satisfaction which can increase customer loyalty. This shows that the national public banking in DKI Jakarta should be able to have an advantage in implementing information technology, owning and implementing good corporate governance (GCG), and have the ability to utilize human resource competency behavior that has a high competency and attitude (Pribadi and Kanai 2011) Mayden and Lado (2007).

## Findings

The national public banking in Jakarta will be able to improve marketing performance if the company has a competitive advantage, while the competitive advantage in the company’s technology will be superior if the company has a reliable information technology system, especially in applications (software) (H2b) and have good corporate governance, especially in the independence (H1d) of implementing the precautionary principles which are supported by human resources especially in the behavior of high competency (H3a).

## Conclusion

Good governance have positive and significant influence on competitive advantage. Transparency, accountability, responsibility, independency, and fairness have positive and significant effects on competitive advantage, but independency has dominant effects on competitive advantage.

Information technology has positive dan significant effects on competitive advantage. Database, software and hardware systems have positive and significant effect on competitive advantage, but software system has dominant effects on competitive advantage.

HR competency has positive and significant effects on competitive advantage. Behavior competency and attitude competency have positive and significant effects on competitive advantage, but behavior competency has a dominant effect on competitive advantage.

Competitive advantage has positive and significant effects on marketing performance.
